# DNA methylome analysis provides evidence that the expansion of the tea genome is linked to TE bursts

**DOI:** 10.1111/pbi.13018

**Published:** 2018-10-15

**Authors:** Lei Wang, Yan Shi, Xiaojun Chang, Shengli Jing, Qunjie Zhang, Chenjiang You, Hongyu Yuan, Haifeng Wang

**Affiliations:** ^1^ Henan Key Laboratory of Tea Plant Biology College of Life Science Xinyang Normal University Xinyang China; ^2^ State Key Laboratory of Ecological Pest Control for Fujian and Taiwan Crops College of Plant Protection Fujian Agriculture and Forestry University Fuzhou China; ^3^ College of Horticulture Fujian Agriculture and Forestry University Fuzhou China; ^4^ Agrobiological Gene Research Center Guangdong Academy of Agricultural Sciences Guangzhou China; ^5^ Guangdong Provincial Key Laboratory of Plant Epigenetics College of Life Sciences and Oceanography Shenzhen University Shenzhen China; ^6^ Department of Botany and Plant Sciences Institute of Integrative Genome Biology University of California Riverside CA USA

**Keywords:** DNA methylation, tea methylome, genome expansion, TE burst

## Abstract

DNA methylation is essential for gene regulation, imprinting and silencing of transposable elements (TEs). Although bursts of transposable elements are common in many plant lineages, how plant DNA methylation is related to transposon bursts remains unclear. Here we explore the landscape of DNA methylation of tea, a species thought to have experienced a recent transposon burst event. This species possesses more transposable elements than any other sequenced asterids (potato, tomato, coffee, pepper and tobacco). The overall average DNA methylation levels were found to differ among the tea, potato and tomato genomes, and methylation at CHG sequence sites was found to be significantly higher in tea than that in potato or tomato. Moreover, the abundant TEs resulting from burst events not only resulted in tea developing a very large genome size, but also affected many genes involved in importantly biological processes, including caffeine, theanine and flavonoid metabolic pathway genes. In addition, recently transposed TEs were more heavily methylated than ancient ones, implying that DNA methylation is proportionate to the degree of TE silencing, especially on recent active ones. Taken together, our results show that DNA methylation regulates transposon silencing and may play a role in genome size expansion.

## Introduction

DNA methylation is a stable epigenetic mark that is widespread among eukaryotic species. Extensive studies have shown that DNA methylation plays an important role in regulation gene expression and transposon silencing (Finnegan *et al*., 2000; Gehring and Henikoff, 2007). Unlike in animals, where methylation often occurs exclusively on CG sequences, in plants, methylation is common in three different contexts: CG, CHG (where H can be A, T or C) and CHH (Cokus *et al*., 2008; Feng *et al*., 2010; Lister *et al*., 2008). The maintenance of CG context methylation is functionalized by methyltransferase 1 (MET1), which is recruited to hemi‐methylated CG sites during DNA replication (Bostick *et al*., 2007). In contrast, CHG methylation is maintained by plant‐specific chromomethylase 3 (CMT3); this methylation is strongly associated with dimethylation of lysine 9 on histone 3 (H3k9me2), and CMT3 acts together with histone methyltransferase and SUVH5/6 to form a self‐reinforcing loop (Jackson *et al*., 2002; Lindroth *et al*., 2001). CHH methylation is established and maintained by CMT2, and like CHG methylation, it is associated with methylation in H3K9me2 regions (Gouil and Baulcombe, 2016). *De novo* establishment of DNA methylation in all three contexts is mediated by the RNA‐directed DNA methylation (RdDM) pathway guided by small RNAs and domains rearranged methyltransferase 2 (DRM2) (Cao and Jacobsen, 2002). Recent studies have showed that RdDM and CMT2 often complement each other. Moreover, CMT2 also plays a role in CHG methylation (Law and Jacobsen, 2010; Stroud *et al*., 2013).

Current knowledge of DNA methylation in plants is primarily based on studies of *Arabidopsis thaliana*, which has a compact genome and is relatively tolerant to significant reductions in DNA methylation. However, most plant genomes are much larger and possess an abundance of transposons (Dodsworth *et al*., 2015). Genome‐wide DNA methylation patterns may vary considerably, even among closely related plant species (Kim *et al*., 2015; Wang *et al*., 2018). Advances in DNA methylation sequencing have permitted the construction of the methylomes of many plant species, often profiled at single‐base resolution (Niederhuth *et al*., 2016). Comparative methylome studies have shown that genome‐wide average DNA methylation levels are positively correlated with genome size and TE contents (Takuno *et al*., 2016; Wang *et al*., 2018); thus, the larger the genome, the higher the DNA methylation level of the DNA.

Tea is one of the world's most important beverage crop, and provides a wealth of health benefits. Analysis of tea genome has revealed that this species experienced a burst event of transposable elements in its evolutionary past, and now possesses one of the largest genomes of all sequenced asteroid species (Wei *et al*., 2018; Xia *et al*., 2017). The availability of the tea genome allows us to explore the DNA methylation landscape at the whole genome level and to determine the relationship between DNA methylation and the abundance of transposons in the tea genome. To better understand the role of DNA methylation in the TE‐abundant tea genome, we present a single‐base resolution DNA methylation landscape of the tea by whole genome bisulphite sequencing. We found that the levels and patterns of DNA methylation are different from those found in other asterids, such as potato and tomato, and that CHG methylation level is significantly higher in the tea genome relative to other plant species. This fact is likely due to the presence of abundant repetitive DNA elements and the large overall size of the genome. In addition, average DNA methylation levels of TEs are reduced as the evolutionary ages increased, suggesting DNA methylation roles on silencing of recent active transposons.

## Results

### DNA methylation pathway‐related genes in the tea genome

Genetic studies of the model plant *Arabidopsis thaliana* have defined many of the key components involved in the DNA methylation pathways, including MET1, DRM2, CMT2, and CMT3 (Law and Jacobsen, 2010; Matzke *et al*., 2015). To comprehensively assess the function of these genes in tea, we searched the tea genome for homologs of *A. thaliana* methylation pathway‐related genes by using BLASTP and the HMM algorithm. We found that most of the DNA methylation pathway‐related genes found in *A. thaliana* are conserved in tea, including MET1, CMT2, CMT3 and DRM2 (Table 1). In addition, at least one copy of homologs of genes involved in the RNA‐directed DNA methylation pathway (RdDM), including SHH1, NRPD1, NRPE1, DMS3 and DCL2, were also found in the tea genome. Furthermore, RNA‐seq data showed that those genes were expressed (Table 1). Taken together, our data suggest that the DNA methylation pathway is functional and conserved in tea.

**Table 1:** Putative DNA methylation pathway genes in tea tree

	Tea tree (*Camellia sinensis*)			
	Name (Arabidopsis)	Length (a.a)*	Orthologs	Expression level (FPKM)
MET1	VIM1,2,3,4,5,6	645	CSA002777.1	2.55959
	MET1,2a,2b,3	1534	CSA018787.1	0.987532
CMT3	SUVH4	624	CSA002812.1	11.2352
	CMT2	1295	CSA032127.1	2.48205
	CMT3	839	CSA030966.1	3.81276
Pol IV recruit	CLSY1/CLSY2	1256	CSA023919.1	11.5655
	SHH1/SHH2	258	CSA015026.1	2.85557
Pol IV	NRPD1	1453	CSA001027.1	4.89804
Pol IV+V	NRPD2/NRPE2	1172	CSA013061.1	3.41522
Pol IV+V	NRPD4/NRPE4	205	CSA033129.1	5.28803
Pol V	NRPE1	1976	CSA002792.1	8.60674
Pol V	NRPE5	222	CSA008802.1	23.2597
Pol V	NRPE9B	114	CSA022741.1	9.89088
Pol V recruit	DRD1	888	CSA033157.1	6.42526
	DMS3	420	CSA003130.1	3.2686
	RDM1	163	CSA033069.1	5.54529
	SUVH2/9	650	CSA033444.1	15.037
RdDM	RDR2	1133	CSA003297.1	5.3044
	DCL1	1910	CSA034540.1	1.34299
	DCL2	1388	CSA001781.1	3.48661
	DCL3	1580	CSA030036.1	10.0673
	DCL4	1702	CSA011120.1	1.0754
	HEN1	942	CSA016903.1	1.51594
	AGO4	924	CSA016856.1	57.3644
	KTF1	1493	CSA021730.1	11.9791
	IDN2	647	CSA020735.1	8.02238
	SUVR2	740	CSA021101.1	2.91624
	DMS4	346	CSA023931.1	10.5941
	UBP26	1067	CSA033342.1	20.4559
	DRM2	626	CSA001531.1	13.0907
	LDL1	844	CSA034653.1	4.18459
	LDL2	746	CSA000647.1	1.91191
	JMJ14	954	CSA018589.1	3.90207
Others	HDA6	471	CSA007759.1	12.977
	RDR6	1196	CSA020358.1	11.412
	MORC6	663	CSA024443.1	4.76682
	DDM1	764	CSA030020.1	2.27703

For each gene family, the length of protein indicates the longest protein in this gene family, and only one ortholog was identified in Tea tree genome.

### Genome‐wide profile of DNA methylation in tea

To explore single‐base resolution of DNA methylation in tea, we performed whole genome bisulphite sequencing (BS‐seq) of young leaf tissue. We used two independent biological replicates and generated 1.3 billion reads in total. BS‐seq reads were mapped to the *Camellia sinensis* var. *assamica* genome (Xia *et al*., 2017), and methyl‐cytosines were identified by BSMAP (Xi and Li, 2009). More than 70% of the total reads were uniquely mapped, yielding an average ~25 coverage sequencing depth of each replicate (Table S1). Meanwhile, approximately 80% of the total cytosines were covered by at least 1 read (Figure S1). A pairwise comparison of the methylation levels of 2 kb bins across the genome revealed that two biological replicates were well correlated in all three‐sequence contexts, indicating high reproducibility and adequate coverage of our replicates (Figure S2).

**Figure 1:**
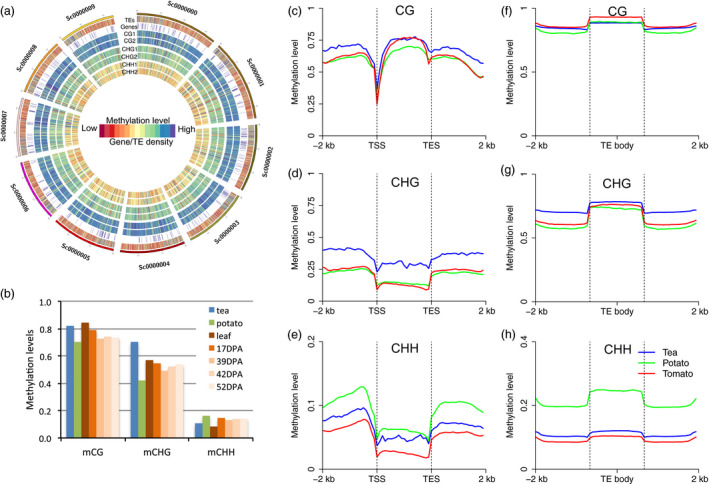
The DNA methylome landscape of tea tree. (a) The landscape of DNA methylation in the first 10 chromosomes of tea. From outer to inner: TE density, gene density, and methylation of CG rep1, CG rep2, CHG rep1, CHG rep2, CHH rep1 and CHG rep2. Blue indicates high methylation levels and high gene/TE density. Red indicates low methylation and low gene/TE density. (b) DNA methylation comparison between tea and other asterids, including potato and tomato (leaf, and fruit ripening stages: 17 days post anthesis (DPA), 39DPA, 42 DPA and 52DPA). (c–e). DNA methylation patterns of gene body and flanking regions in the CG, CHG and CHH sequence contexts. (f–h) DNA methylation patterns of transposon and flanking regions in the CG, CHG and CHH sequence contexts. In all cases, −2 kb indicates the 2000 bp upstream from transcriptional start sites (TSS), and 2 kb indicates the 2000 bp downstream from transcriptional end sites (TES). Upstream regions, gene bodies and downstream regions were divided into 20 proportionally sized bins to draw the metaplot.

The genome‐wide landscape of DNA methylation of the first ten scaffolds are shown in Figure 1a. Consistent with Figure S2, we found that the DNA methylation patterns of both replicates were like one another along the entire length of the scaffolds, and that TE density was correlated with DNA methylation level. The genome‐wide average methylation of the CG, CHG and CHH contexts were 82%, 70% and 10%, respectively; these levels were much higher than those found in potato at CG and CHG sites (Figure 1b). We also observed that the position‐wise frequency of CG and CHG methylation showed bimodal distribution; this pattern was seen in both potato and tomato (Figure S3A). Moreover, highly methylated CHG sites (i.e. those with methylation levels >75%) were found in greater abundance than in potato and tomato. However, the proportion of methyl‐cytosines of the different contexts were similar among the three asterids genomes, and very different from the proportions found in other plant lineages, implying that the proportion of methyl‐cytosines is lineage‐specific (Figure S3B). The DNA methylation of symmetric sites (CG and CHG) was also found to be highly correlated highly in *Arabidopsis*, but the weak correlation of the CG and CHG sites has also been observed in a closely related relative, *Arabis alpina* (Willing *et al*., 2015). In tea, we also observed a weaker correlation of DNA methylation between two strands at CG and CHG sites than was found in potato and tomato (Figure S4). This suggests that the maintenance of both CG and CHG methylation is relatively weak. In addition, we found that CHG methylation was significantly higher than in both potato and tomato, and was comparable to other plants such as maize and Norway spruce (Ausin *et al*., 2016; Niederhuth *et al*., 2016). Recent studies have confirmed that the proportion of the genome composed of transposable elements and methylation levels of symmetrical contexts are both positively correlated with genome size (Takuno *et al*., 2016; Wang *et al*., 2018), partly explaining the high CHG methylation levels found in the tea genome.

Since genome‐wide average DNA methylation was very different among tea and other two asterids examined (potato and tomato), we compared the DNA methylation levels of genic and TE regions in tea, potato and tomato, and to investigate the relative patterns of DNA methylation in gene and TE regions in tea compared to potato and tomato. We found that both gene and TE methylation patterns were similar to patterns found in potato and tomato (Figure 1c–h), as well as in other plants (Feng *et al*., 2010; Niederhuth *et al*., 2016; Wang *et al*., 2015, 2018). As expected, we found that CHG methylation was higher in both gene body and TE regions compared to those in potato and tomato (Figure 1d,g), which is consistent with genome‐wide average DNA methylation comparisons (Figure 1b). In addition, we found that CHH methylation was similar in tea and tomato in both gene and TE regions, but was much higher in potato, which confirms the findings of a previous study (Figure 1e,h; Wang *et al*., 2018). Taken together, CHG methylation was the most different methylation comparing to other asterids plants, and might be related to abundant transposable elements contained in the tea genome.

### Association between DNA methylation and gene transcription

**Figure 2:**
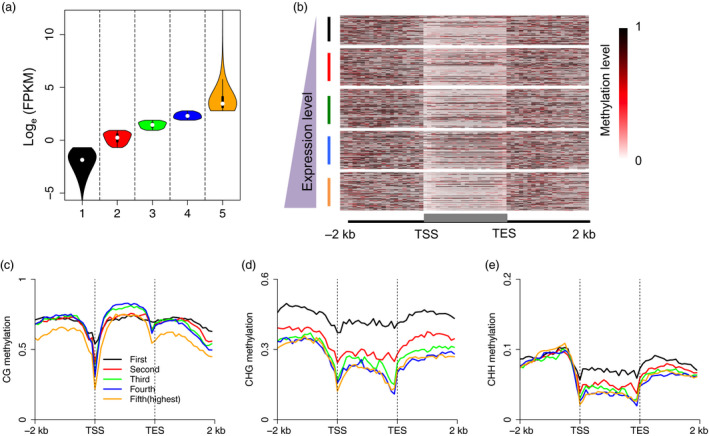
Association between DNA methylation and gene expression. (a) Genes were divided into five groups according to their expression levels. (b) CG methylation levels across gene body and flanking regions of the five groups of genes. (c–e) Association between gene expression and DNA methylation in the CG, CHG and CHH sequence contexts. Genes with FPKM value <0.5 were sorted into the first group, and all other genes were proportionally divided into the other four groups by their gene expression level. This meant that the fifth group of genes contained the highest expressed genes. Each upstream 2 kb, gene body, and downstream 2 kb region was divided proportionally into 20 bins, and average methylation levels were estimated for each bin.

To assess the relationship between DNA methylation and gene transcription, we first grouped genes into five groups according to their expression levels (Figure 2a). A positive correlation was observed between gene body CG methylation and gene expression levels (Figure 2b,c), but not between CHG and CHH methylation and expression (Figure S5). In addition, consistent with previous studies in other plant species (Li *et al*., 2012; Wang *et al*., 2015; Zemach *et al*., 2010; Zhang *et al*., 2006), moderately highly expressed genes were the most heavily methylated in the CG sequence context (i.e. the third and fourth groups of genes in this study), and not the group of genes showing the lowest expression (i.e. the first group of genes) or the group showing the highest expression (i.e. the fifth group of genes) (Figure 2c). Unexpectedly, we observed that promoter and downstream CG methylation in the fifth group of genes was hypo‐methylated relative to the methylation levels of the other groups of genes. In fact, the other four groups of genes showed no differences in both promoter and downstream CG methylation, implying that methylation of flanking and gene regions act together to regulate gene expression. Unlike CG methylation, a negative relationship was found between non‐CG methylation and gene expression, and the lowest expressed genes possessed the highest methylation levels in gene body regions (Figure 2d,e).

**Figure 3:**
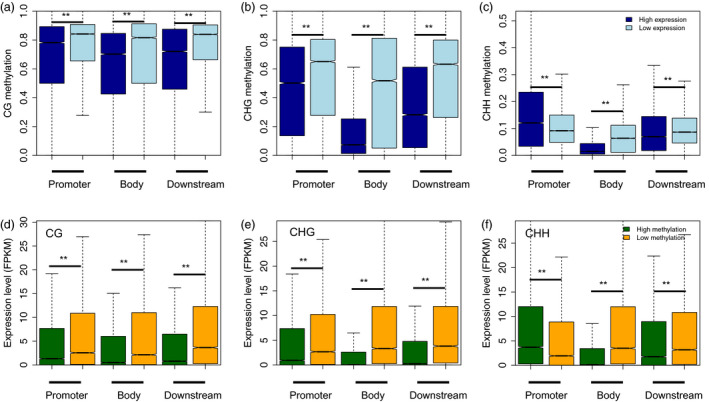
Association between gene expression and methylation at different genic regions. Comparison of methylation between lowly and highly expressed genes for the CG (a), CHG (b) and CHH (c) sequence contexts for each genic regions (i.e. promoter (upstream 2 kb), gene body and downstream 2 kb). Also shown are: a comparison of the expression levels between lowly‐ and highly methylated genes for the CG (d), CHG (e) and CHH (f) sequence contexts of each genic regions. Highly‐ and lowly expressed genes were defined as the one‐third of genes with the highest and lowest expression levels, respectively. Highly‐ and lowly methylated genes were defined as the one‐third of genes with the highest and lowest methylation levels with respect to each genic region, respectively. *P*‐values were calculated by Mann–Whitney tests, and double stars indicate a *P*‐value <0.001.

To further examine the relationship between DNA methylation and gene expression in different sequence contexts (CG, CHG and CHH) and in different genomic regions (i.e. in promoters, gene regions, downstream regions, see the Methods section for further explanation), we compared the methylation levels of lowly‐ and highly expressed genes in the CG, CHG and CHH sequence contexts in different genomic regions. Our results showed that lowly expressed genes were methylated to a significantly higher degree in the CG and CHG contexts of promoter regions (Mann–Whitney test, *P*‐value <0.001), but not in the CHH context in these regions (Figure 3a–c). In addition, the DNA methylation levels of gene body and downstream regions were also higher in lowly expressed genes compared to highly expressed genes. Conversely, we also classified genes into highly‐ and lowly methylated genes, and assessed whether these genes differed in expression level. We also included the methylation levels of different genomic regions in this analysis. Consistent with our results described above, we found that highly methylated genes were always expressed at significantly lower levels than genes that were lowly methylated. This was true for all genic regions in the CG and CHG sequence contexts (Mann–Whitney test, *P*‐value <0.001), but not for highly methylated promoter regions in the CHH sequence context (Figure 3d–f). Taken together, our data suggest that the relationship between DNA methylation and gene expression is complicated, and is also affected by sequence context and genomic region.

### Intragenic TEs induced increased DNA methylation of orthologous genes in the tea genome

**Figure 4:**
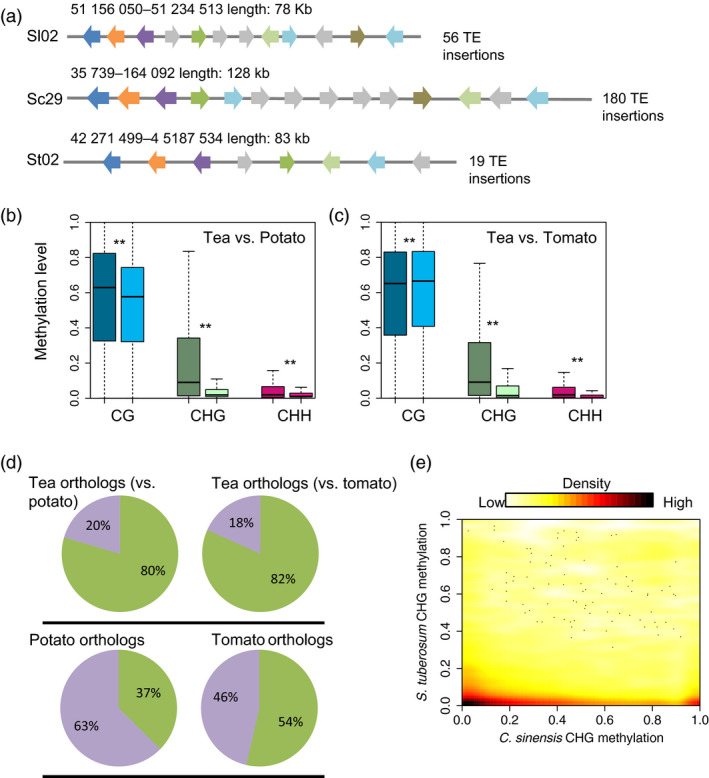
Comparisons of orthologous gene pairs in tea, potato and tomato. (a) A comparison of collinear regions of tea, tomato and potato. Orthologous gene pairs are indicated by the same colours, and the grey colour indicates non‐homologues genes. DNA methylation comparisons of orthologous gene pairs between tea and potato (b), and tea and tomato (c) are shown for all three sequence contexts. Double stars indicate Mann–Whitney test *P*‐value <0.001. Dark colours indicate the CG (blue), CHG (green) and CHH (red) sequence contexts in tea, and light colours indicate the same in potato and tomato. (d) Pie charts illustrating the percentage of TE‐associated (green) and non‐TE associated (purple) orthologous genes in tea and potato, tea and tomato, and their orthologous copies in potato and tomato, respectively. TE‐associated genes are defined as genes that overlap with TEs. (e) Distribution of CHG methylation levels of 20 990 orthologous gene pairs between tea and potato.

The striking differences of DNA methylation levels between tea and other two asterids studied here, potato and tomato, are probably due to difference in TE contents in their respective genomes. We also found that, relative to potato and tomato, in tea both gene and TE regions were hypermethylated in the CHG sequence context (Figure 1d,g). To assess the effect of TE abundance on genome structure and DNA methylation, we compared the genomes of tea, potato and tomato and explored the collinear regions in these three genomes. We found that when comparing homologous regions of the tea genome always harboured more TE insertions than the other two species, resulting in tea genome reaching a larger size. Figure 4a shows collinear regions in tea, potato and tomato: in total, we found 128 kb homologous regions in tea, but only 83 kb, and 78 kb homologous regions in the genomes of potato and tomato, respectively. Detailed examination revealed 180 TE insertions in the homologous regions of the tea genome, but only 19 and 56 TE insertions in the genome of potato and tomato, respectively. To investigate the effect of TE insertions in orthologous genes of collinear regions among these three plants, we compared the DNA methylation levels of orthologous genes in tea, potato and tomato in the three sequence contexts. As expected, higher DNA methylation levels were observed in tea than in potato and tomato, and this was especially true in the CHG sequence context (Mann–Whitney test, *P*‐value <0.01) (Figure 4b,c). This finding was consistent with the genome‐wide DNA methylation and metaplot analyses of gene and TE regions, in which CHG methylation was also found to be much higher in tea than in potato or tomato (Figure 1b,d,g). We know that DNA methylation could spread from TE boundaries to closed gene regions, and that this generally results in increased DNA methylation levels of protein‐coding genes near to TE regions (Gent *et al*., 2013).

Next, we compared the proportions of TE‐associated genes in tea versus potato or tomato separately. We defined TE‐associated genes as those protein‐coding genes that overlap with TEs in their flanking 2 kb regions. Strikingly, we found more TE‐associated genes in tea than in potato or tomato (80% vs 37% in tea and potato, 82% vs 54% in tea and tomato) (Figure 4d). Interestingly, we found that many genes encoding secondary metabolites were enriched in TE‐associated genes. These genes included genes involved in caffeine metabolism (e.g. IMPDH, TCS, SAMS and AMPDA), flavonoid metabolism (e.g. 4CL, PAL, C4H, F3'H and FLS) and theanine metabolism (e.g. TS).

Recent studies have verified that the DNA methylation levels of homologous genes in different plants are often very similar to one another. This is especially true of CG and CHG methylation (Niederhuth *et al*., 2016; Takuno and Gaut, 2013; Wang *et al*., 2018), However, we did not observe this phenomenon in our comparison of tea with potato and tomato. On the contrary, we found that no correlation between tea and potato orthologs with respect to CHG methylation levels (Figure 4e). This finding was also true of comparisons between tea and tomato in all sequence contexts (Figure S6). In summary, our data imply that high levels of TE insertions can not only result in genome size enlargement, but also result in higher levels of DNA methylation than in closely related plants with low levels of TE insertions.

### Reduced DNA methylation levels of TEs along evolutionary ages

**Figure 5:**
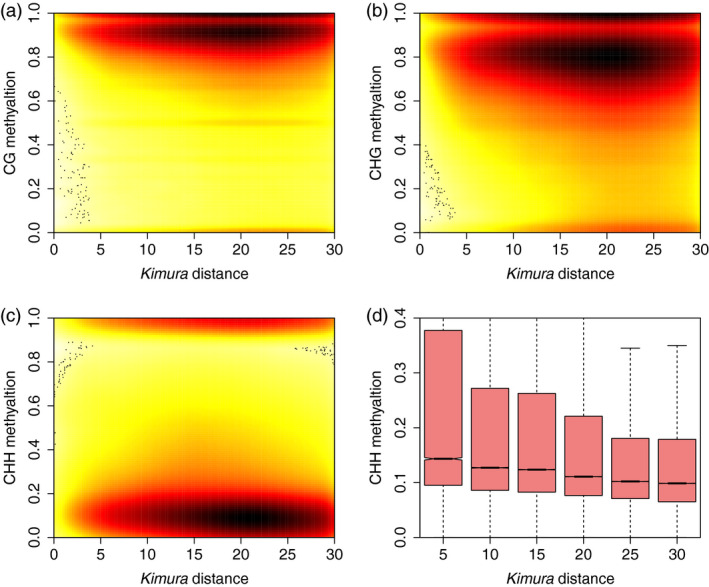
Association between the age of transposons and DNA methylation. A scatterplot showing the negative correlation between Kimura distance and CG (a), CHG (b) and CHH (c) DNA methylation. Kimura distance was used as a proxy for transposon age. (d) Boxplot showing the reduced CHH methylation of transposons as the Kimura distance increased.

Recent studies have shown that recently duplicated genes were hypermethylated relative to older ones, and that duplicates show decreases in DNA methylation as evolutionary time passes (Keller and Yi, 2014). To explore the relationship between DNA methylation and TE insertion time, Kimura distances (Kimura, 1980) were calculated for all TE copies found in each family to estimate the age of TE insertion. Like the Ks value (synonymous substitution divergence), which estimates the age of duplicated genes, Kimura distance estimates are based on TE similarity: the presence of copies of TEs that are similar (i.e. a low Kimura distance) indicates recent insertion, while the presence of unique or divergent copies of TEs (i.e. a high Kimura distance) indicates that these TEs originate from relatively ancient transposition events. Consistent with a recent study regarding TE expansion in the tea genome (Xia *et al*., 2017), we observed a TE burst event around 20 Kimura distance (Figure S7). Like duplicated genes in human genome, we found a negative correlation between the age of TE insertion and DNA methylation level in all three sequence contexts (Figure 5a–c). Due to a TE burst event in the past (Kimura distance ~20), we found many TE copies with a Kimura distance of approximately 20. Among the three sequence contexts, the CHH methylation levels of different TEs showed greater reductions through evolutionary time (Figure 5d) than the CG and CHG methylation levels (Figure S8A,B). This implies that the RdDM pathway plays an important role in silencing recently active TEs but gradually loses its effectiveness in silencing more ancient ones.

## Discussion

In this study, we provide the first high‐resolution genome‐wide DNA methylation map of the tea genome. Tea has numerous healthy and medicinal benefits for humans due to its abundance of secondary metabolites (Wei *et al*., 2018; Xia *et al*., 2017). Given the important role that DNA methylation plays in the control of gene expression and transposon silencing, our data serve as an important resource for the scientific and agronomic community.

In present study, we first identified DNA methylation related genes and found most of them have at least one copy in tea tree genome, and RNA‐seq data showed relatively high expression level, which is consistent to our RT‐PRC results (Figure S9 and Table S2). These data suggest DNA methylation pathway is conserved and functional in tea tree genome. Comparative analysis of the methylomes of tea, potato and tomato revealed that a considerable proportion of the variation of DNA methylation occurs between species. The greatest difference in DNA methylation between these species was observed in the CHG sequence context, one hypothesis is that tea may have a more robust maintenance methylation mechanism for CHG sites than potato and tomato. But weak DNA methylation correlations between the two strands of DNA were found at symmetrical CG and CHG sites in the tea genome, implying that the methylation maintenance pathway at CG and CHG sites are not as robust in tea as they are in potato and tomato. Tea also has higher TE contents in general than in potato and tomato, and recent studies have shown that both CG and CHG methylation levels are significantly positively correlated with TE content and genome size (Niederhuth *et al*., 2016; Takuno *et al*., 2016; Wang *et al*., 2018). Thus, the other hypothesis is that the superabundance of TEs presented in the tea genome has resulted in higher CHG methylation than is present in potato or tomato. We also observed that CHG methylation levels in both gene and TE regions were much higher in tea than in potato and tomato, which also suggests an effect of genic TE insertions generated from a TE burst event. Furthermore, we also observed that many genes encoding secondary metabolism were TE‐associated genes, such as caffeine, theanine and flavonoid metabolic pathway genes. This implies that epigenetic modifications such as DNA methylation may affect tea flavour by regulating secondary metabolic pathways.

Examination of the DNA methylation of TEs from different evolutionary periods revealed that recent active TEs are heavily methylated, while methylation is reduced in older TEs. This indicates that in tea the average DNA methylation levels of TEs are negatively correlated with evolutionary time, as measured by Kimura distance. This finding is consistent with observations of duplicated genes in the human genome (Keller and Yi, 2014). Taken together, this study demonstrates that heavy CHG methylation is present in the tea genome (along with abundant TE contents), and that the variation in DNA methylation levels occurs through evolutionary time. Recently, non‐canonical DNA modifications have been identified and characterized in many eukaryotes (Greer *et al*., 2015; Liang *et al*., 2018; Zhou *et al*., 2018), such as N^6^‐methyladenine (also referred as 6 mA). In plants, 6 mA was also identified in Arabidopsis and rice, and both plants showed that 6 mA was associated with gene expression, suggesting its important roles on plant development. In rice, 6 mA also was correlated with 5 mC at CG sites in gene body regions, and is complementary at CHH site of transposon regions (Liang *et al*., 2018; Zhou *et al*., 2018). Therefore, future studies on DNA methylation should combine with more methylation modifications to comprehensively study their regulation on gene expression. Taken together, this study may help us to understand the extent of DNA methylation variation observed among plants, and tea in particular.

## Experimental procedures

### Kimura distance calculation and estimation of TE burst event

Kimura distance between genome copies and a consensus sequence from a TE library were determined by using the RepeatMasker pipeline. The rates of transitions and transversions were calculated on alignments and transformed to Kimura distance (Kimura, 1980) using the following equation: *K* = −1/2 ln(1−2*p*−*q*)−1/4 ln(1−2*q*). Here, *q* is the proportion of sites with transversions and *p* is the proportion of sites with transitions.

### BS‐seq data analysis

For tomato BS‐seq data (AC cultivar), we used public data (SRA accession SRR046092) from leaf and fruit of different ripening stages, for example, 17 DPA (immature), 39 DPA (mature green), 42 DPA (breaker) and 52 DPA (red ripe) (Zhong *et al*., 2013). For potato BS‐seq data (Atlantic cultivar), we downloaded data from GEO database (accession GSE86983; Wang *et al*., 2018). We first mapped BS‐seq reads to reference genome of tea, potato and tomato using BSMAP v2.90 (Xi and Li, 2009). Only uniquely mapped reads were kept for further analysis. Mapped reads were permitted four mismatches per 100‐bp length. Methylation levels were calculated as weighted methylation level (Schultz *et al*., 2012). Promoter methylation levels were calculated as the average methylation levels of the DNA sequence starting at the transcription start site and counting upstream for 2000 bp. Downstream methylation levels were calculated as the average methylation level of the DNA sequence starting at the transcription end site and counting downstream for 2000 bp.

### RNA‐seq data analysis

For RNA‐seq data, we first mapped clean reads to the tea reference genome by tophat v2.1.0, (Trapnell *et al*., 2009). We kept only uniquely mapped alignments. Expression values were quantified by Cufflinks with FPKM (fragments per kilobase per million mapped reads) with default parameters (Trapnell *et al*., 2012).

### Real‐time PCR validation

Total RNA for real‐time PCR analysis was extracted from tea leaf that was the same stage for RNA‐seq following the cetyltrimethylammonium bromide (CTAB) protocol. RNA quality and quantity were each assessed, respectively, by Agilent 2100 BioAnalyzer (Agilent Technologies, Santa Clara, CA) and NanoDrop 2000 (Thermo Scientific, Waltham, MA). cDNA was synthesized from the total RNA using SuperScript II Reverse Transcriptase system (Invitrogen, Carlsbad, CA) with oligo(dT) following the manufacturer's instructions. The primers (Table 1) for real‐time PCR were designed using NCBI Primer‐BLAST tools according to cDNA sequences. Reactions were carried out on StepOnePlus Real‐Time PCR System (Applied Biosystems, Foster City, CA) using three‐step cycling conditions of 95 °C for 1 min, followed by 40 cycles of 95 °C for 15 s, 60 °C for 30 s and 72 °C for 30 s. After the amplification steps, the melting curve was determined for each primer pair at a final stage of 15 s at 95 °C, 15 s at 60 °C and 15 s at 95 °C to verify the presence of only one specific product. Relative quantification was performed with the 2‐ΔΔCT method. Teaβ‐actin gene (Forward primer: 5′‐GCCATCTTTGATTGGAATGG‐3′, Rverse primer: 5′‐GGTGCCACAACCTTGATCTT‐3′) was used as reference for messenger RNA (mRNA) expression. The Pearson correlation analysis of RNA‐seq and qRT‐PCR was performed by R software (www.r-project.org).

### Homologous gene and collinear regions identification

Homologous genes of tea tree genome were identified by MCScanX (Wang *et al*., 2012), which is an algorithm that can simultaneously scan multiple genomes to identify homologous chromosomal regions and subsequently align these regions by using genes. Collinear regions among tea, potato and tomato were also identified by MCScanX with default parameters, and orthologous genes pairs were extracted from collinear regions.

### Correlation analysis of methylation data

Correlation between biological replicates of BS‐seq was calculated as methylation levels of total Cs in 2000 bp regions. First, tea tree genome was splited into 2000 bin bins, and methylation levels were calculated as the average #C/(#C+#T) for all cytosines in each bin, #C is the number of C reads and #T is the number of T reads. Then, Pearson correlation coefficients were calculated between the two biological replicates.

Correlation analysis between orthologous gene pairs of tea and tomato/potato was performed by corr.test() of R project. DNA methylation level was first calculated of each gene in tea, tomato, and tomato of CG, CHG and CHH sequence contexts. Then, smooth scatter plots were conducted by smoothScatter() of R project (www.r-project.org).

For correlation between DNA methylation and kimura distance, the DNA methylation level of each TE was calculated by custom Perl script, and kimura distance were extracted from RepeatMasker pipeline.

### Metaplot analysis

For metaplot analysis, each gene body region was proportionally divided into 20 bins. Both upstream and downstream 2000 bp regions were also divided into 20 bins (i.e. the length of each bin is 100 bp), separately. Then, the average level of DNA methylation of each bin was calculated for all genes and plotted by R script.

## Accession numbers

The data reported in this paper were deposited in the Gene Expression Omnibus (GEO) database (accession number GSE119992).

## Author contributions

H.W. and H.Y. designed the research; H.W., L.W., Y.S. and X.C. performed the research and analysed the data; S.J. and X.C. collected the data; Q.Z. and C.Y. provided the tools; H.W. wrote the paper.

## Conflicts of interest

The authors declare that they have no conflicts of interest.

## Supporting information


**Figure S1** BS‐seq coverage shown as the proportion of cytosines that were covered by at least ‘X’ reads.
**Figure S2** Correlation analysis between replicates in the CG, CHG and CHH sequence contexts.
**Figure S3** DNA methylation pattern comparison between tea and other plants.
**Figure S4** DNA methylation correlations between Watson and Crick strand in CG and CHG symmetrical sequence contexts in the tea, potato and tomato genomes.
**Figure S5** DNA methylation levels of gene body and flanking regions for five group genes.
**Figure S6** Distribution of DNA methylation levels of orthologous gene pairs between tea and tomato/potato.
**Figure S7** Distribution of Kimura distances of different types of transposons.
**Figure S8** Reduced DNA methylation levels of transposons over evolutionary time.
**Figure S9** Expression correlation between RT‐PCR and RNA‐seq of 37 DNA methylation pathway related genes.

**Table S1** Summary of BS‐seq reads mapping.
**Table S2** The qRT‐PCR primers and 2^−ΔΔCT^ value.
