# Supplementary material for: DNA methylome analysis provides evidence that the expansion of the tea genome is linked to TE bursts
Source: Plant Biotechnol J. 2018 Oct 15;17(4):826–35. doi: 10.1111/pbi.13018 (PMC6419580; doi:10.1111/pbi.13018)
Supplement: Supplementary file 1 — Figure S1 BS‐seq coverage shown as the proportion of cytosines that were covered by at least ‘X’ reads. Figure S2 Correlation analysis between replicates in the CG, CHG and CHH sequence contexts. Figure S3 DNA methylation pattern comparison between tea and other plants. Figure S4 DNA methylation correlations between Watson and Crick strand in CG and CHG symmetrical sequence contexts in the tea, potato and tomato genomes. Figure S5 DNA methylation levels of gene body and flanking regions for five group genes. Figure S6 Distribution of DNA methylation levels of orthologous gene pairs between tea and tomato/potato. Figure S7 Distribution of Kimura distances of different types of transposons. Figure S8 Reduced DNA methylation levels of transposons over evolutionary time. Figure S9 Expression correlation between RT‐PCR and RNA‐seq of 37 DNA methylation pathway related genes. [file PBI-17-826-s002.pdf]

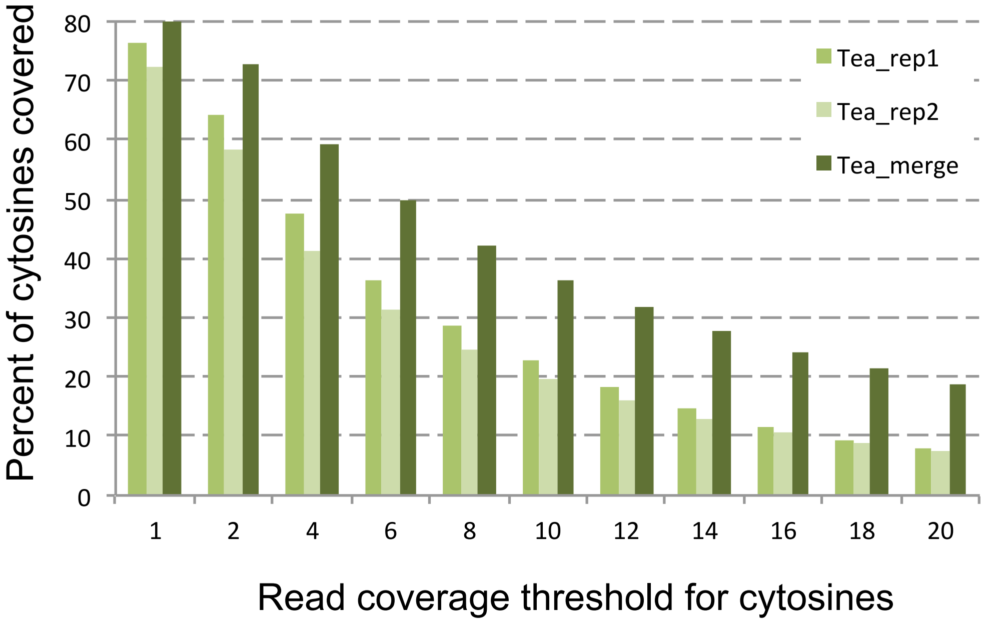

Figure S1. BS-seq coverage shown as the proportion of cytosines that were covered by at least 'X' reads. For example, about 80% of cytosines were covered by at least 1 read in each replicate.

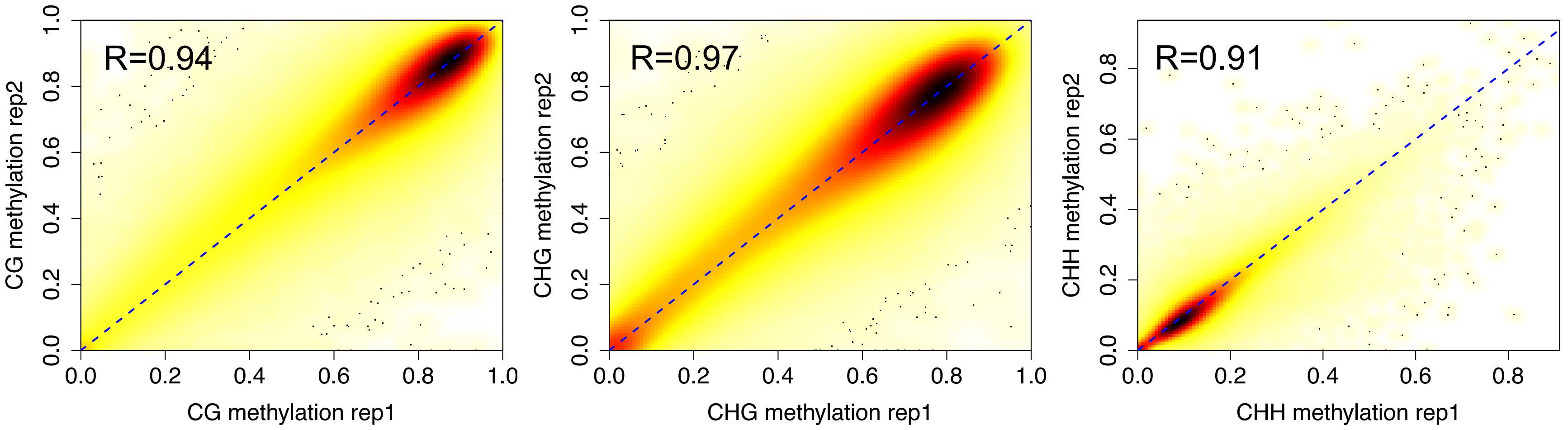

Figure S2. Correlation analysis between replicates in the CG, CHG, and CHH sequence contexts. Methylation levels were calculated for each 2000 bp bin along chromosomes, and the correlation coefficient was estimated between two biological replicates in the CG, CHG, and CHH sequence contexts.

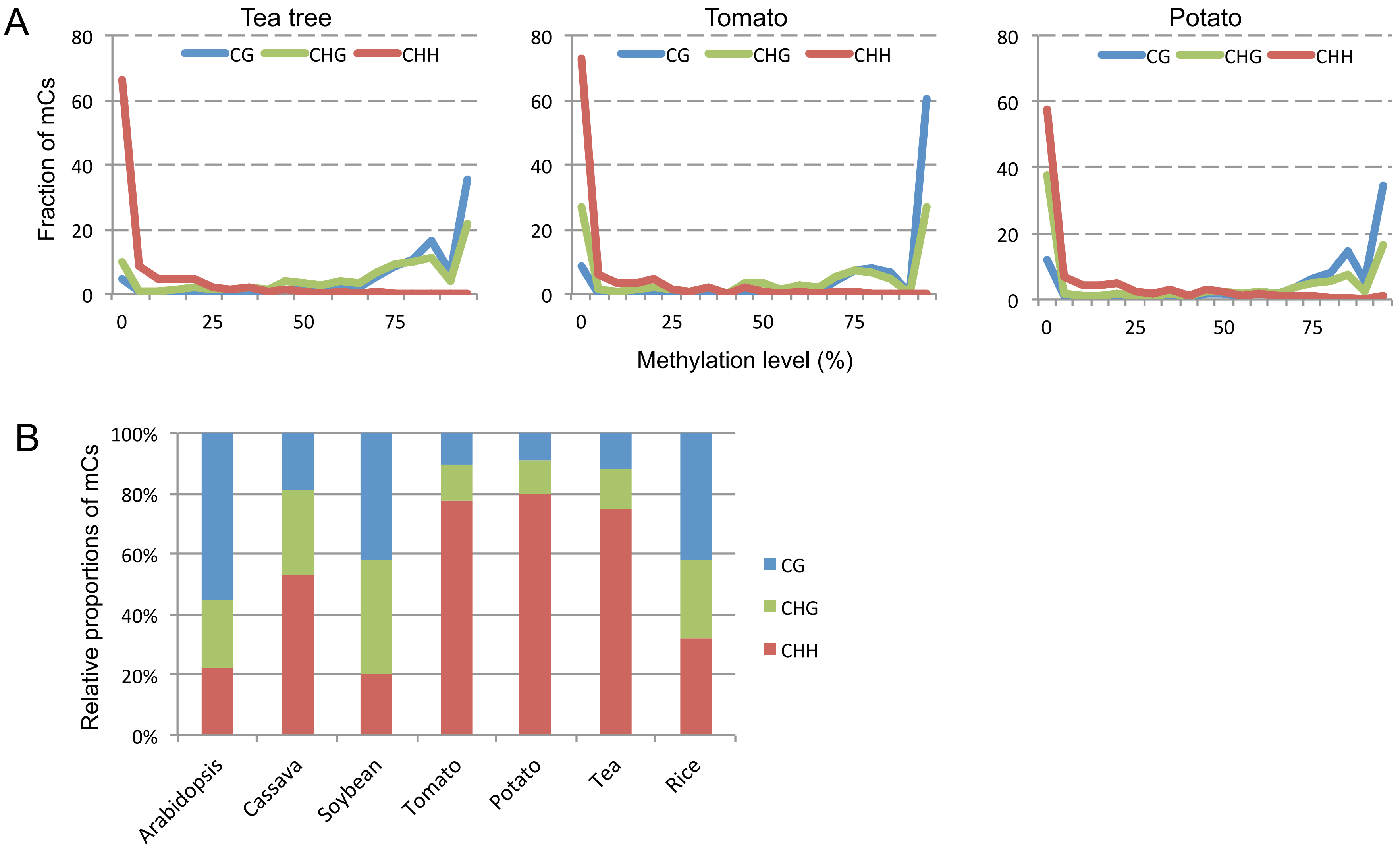

Figure S3. DNA methylation pattern comparison between tea and other plants. (A) Distribution of the methylation level of mCs in each sequence context of tea, tomato, and potato. (B) Relative proportions of mCs in each sequence context among selected plants. Only mCs covered by more than 3 reads were counted and used to calculate weighted methylation level.

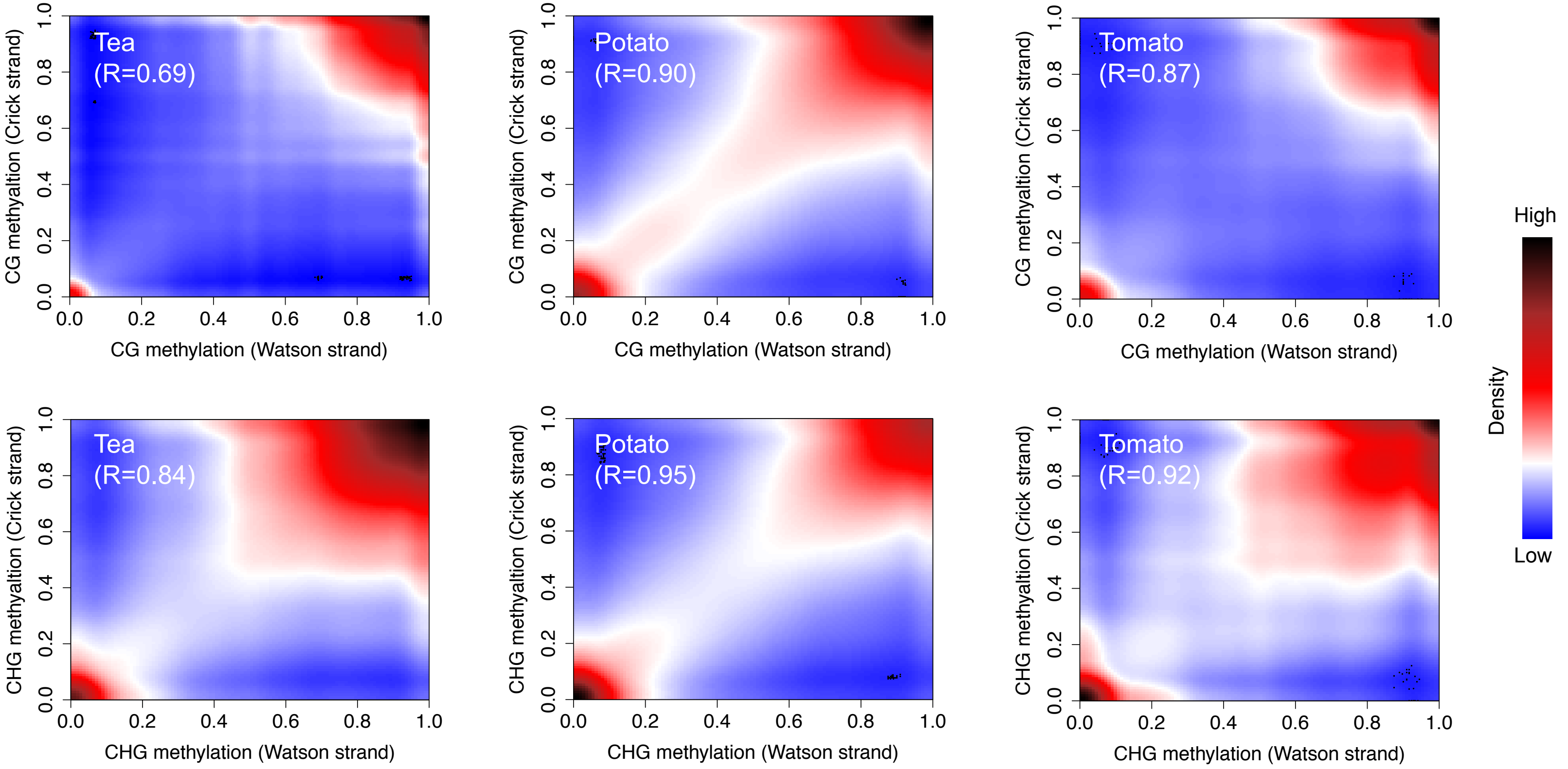

Figure S4. DNA methylation correlations between Watson and Crick strand in CG and CHG symmetrical sequence contexts in the tea, potato, and tomato genomes. Correlation coefficient (R) was calculated as Pearson's correlation coefficient.

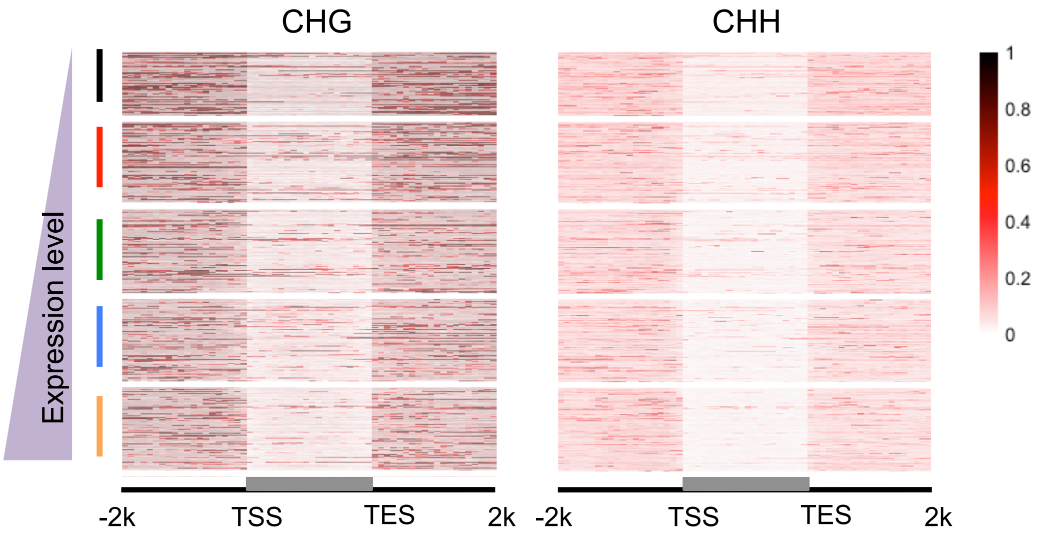

Figure S5. DNA methylation levels of gene body and flanking regions for five group genes. CHG and CHH methylation levels across gene body and flanking regions for five group genes. Genes with FPKM value < 0.5 were classified as members of the first group, and the other genes were proportionally divided into other four groups by methylation level. This meant that the first group of genes contained the lowest expressed genes and the fifth group of genes were the highest expressed genes. Each genic region—i.e. the 2kb upstream flanking region, gene body, and the 2kb downstream flanking region—were proportionally divided into 20 bins, and average methylation levels were estimated for each bin.

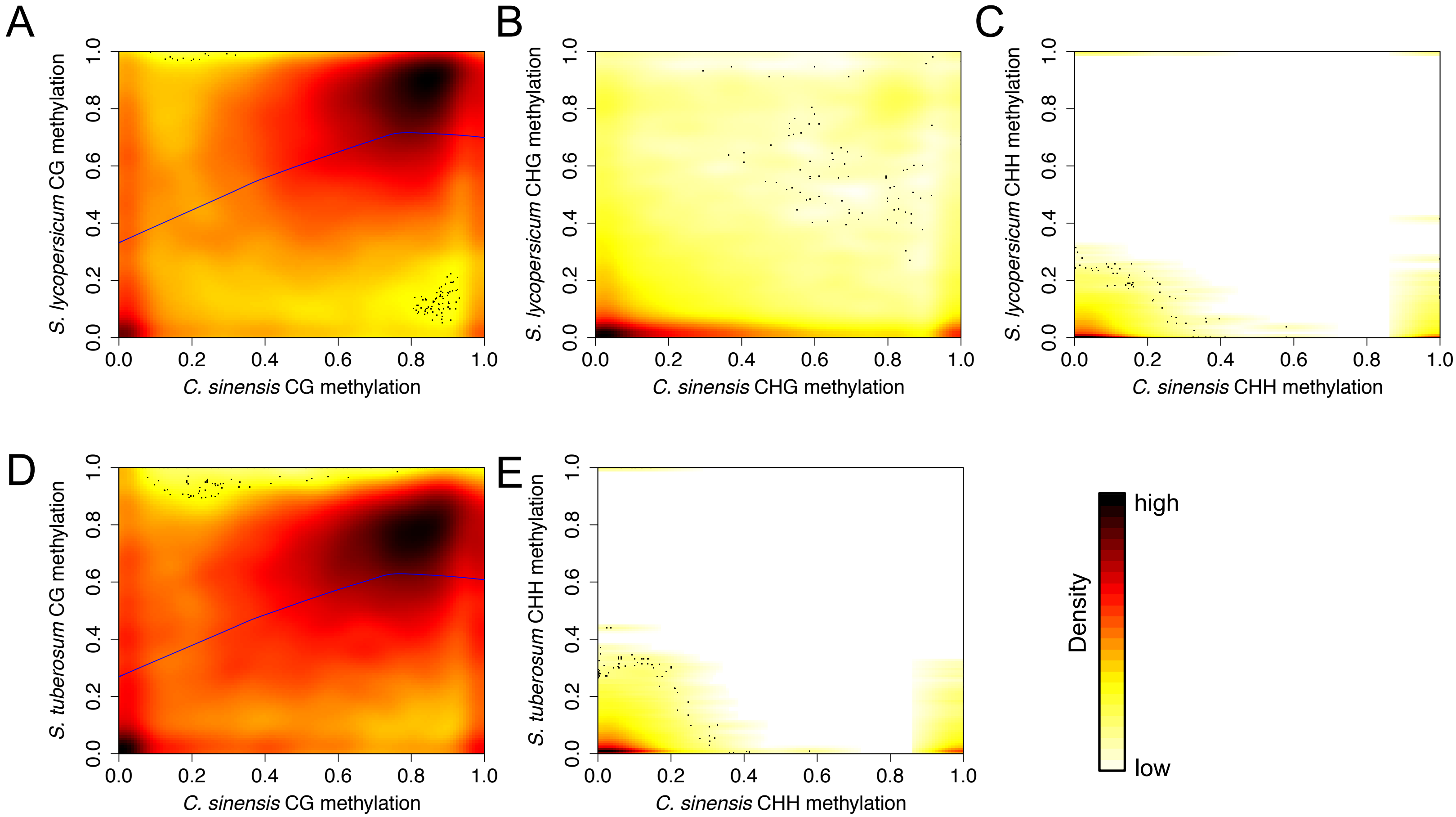

Figure S6. Distribution of DNA methylation levels of orthologous gene pairs between tea and tomato/potato. DNA methylation of orthologous gene pairs between tea and tomato for of CG (A), CHG (B), and CHH (C) sequence contexts. Also shown are the DNA methylation levels of orthologous gene pairs between tea and tomato for the CG (D) and CHH (E) sequence contexts.

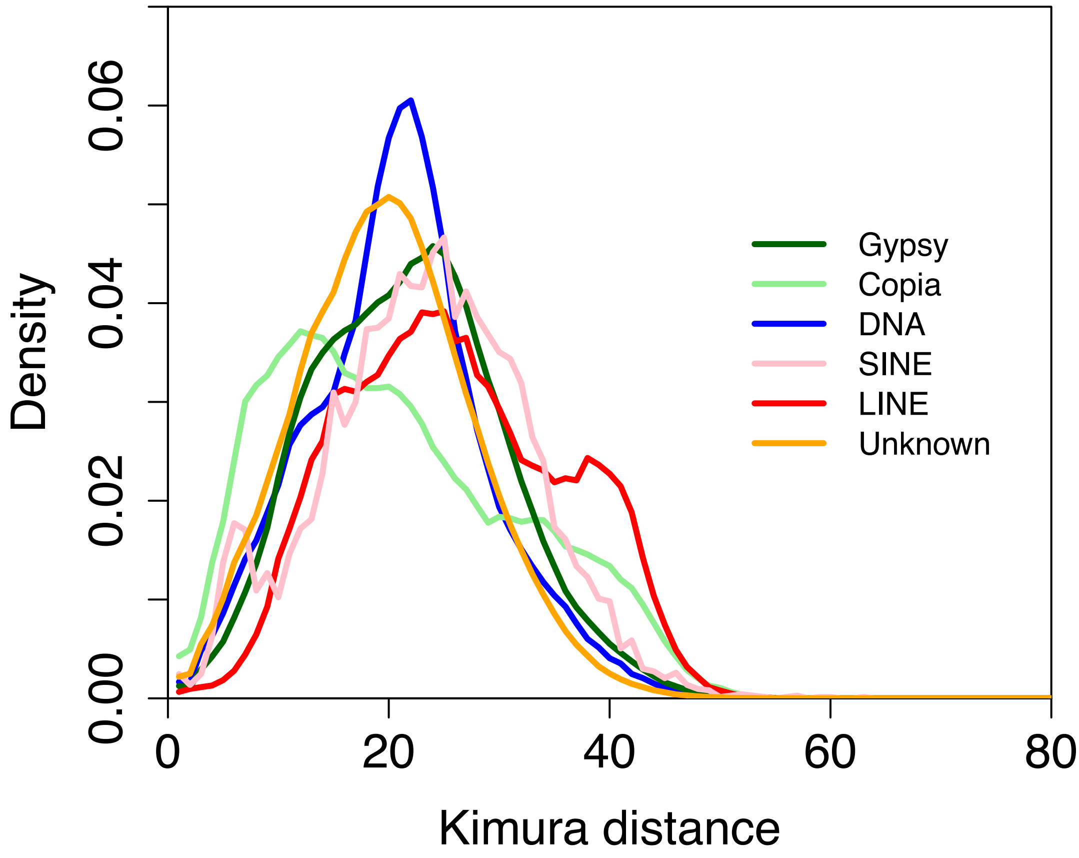

Figure S7. Distribution of Kimura distances of different types of transposons. Kimura distances represent the percentages of substitutions in matching regions compared to the consensus sequences. Kimura distances are estimated by RepeatMasker. The larger the Kimura distance between TEs and their consensus sequence, the older they likely are.

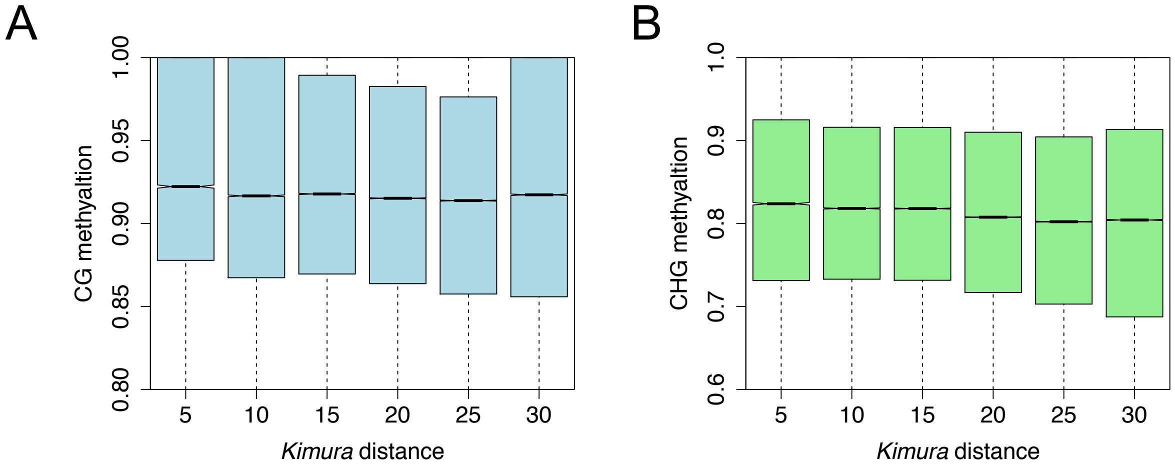

Figure S8. Reduced DNA methylation levels of transposons over evolutionary time. (A) Boxplot showing the reduced CG methylation level of transposons as the Kimura distance increased. (B) Boxplot showing the reduced CHG methylation levels in transposons as the Kimura distance increases.

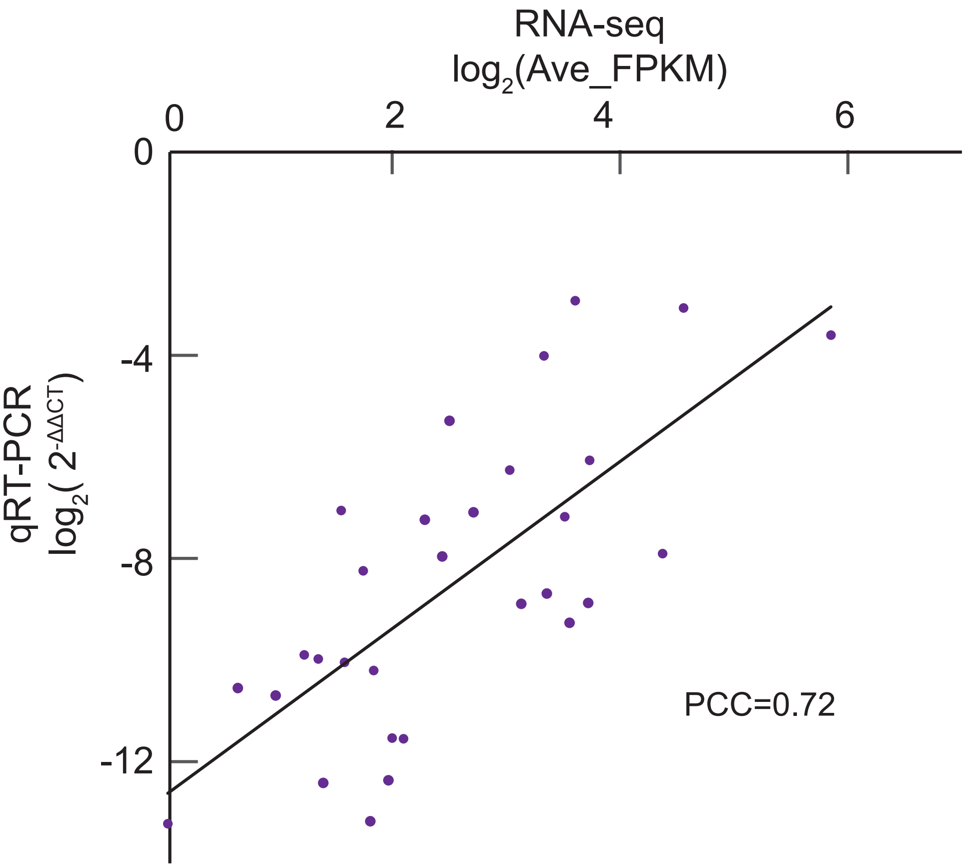

Figure S9. Expression correlation between RT-PCR and RNA-seq of 37 DNA methylation pathway related genes.
