# Supplementary material for: DNA methylome analysis provides evidence that the expansion of the tea genome is linked to TE bursts
Source: Plant Biotechnol J. 2018 Oct 15;17(4):826–35. doi: 10.1111/pbi.13018 (PMC6419580; doi:10.1111/pbi.13018)
Supplement: Supplementary file 2 — Table S1 Summary of BS‐seq reads mapping. Table S2 The qRT‐PCR primers and 2−ΔΔCT value. [file PBI-17-826-s001.pdf]

Table S1. Summary of BS-seq reads mapping.

Sample	Total reads	Uniquely Mapped	Mapped ratio (%)	mCG	mCHG	mCHH	Conversion rate	Coverage
Leaf_rep1	692,216,842	504,815,337	72.93	82.27%	70.13%	10.49%	99.81%	25.06
Leaf_rep2	664,342,280	468,145,810	70.47	82.33%	70.32%	9.69%	99.87%	23.24

Table S2. The qRT-PCR primers and $2^{-\Delta\Delta CT}$ value

Gene	Forward primer (5'-3')	Rverse primer (5'-3')	$2^{-\Delta\Delta CT}$	Ave_FPKM
CSA022741.1	CCAGGAGGTTGCCGATAACA	TCGCCTGGAAGAAAACTGCT	0.062054	9.89088
CSA033157.1	GTGAAGGGTTGGACTCTGGG	CGAGCATCTGGGGAAGTGTT	0.007335	6.42526
CSA003130.1	TTACTGGGCAGAGAAAGGCG	ATATCCTCTTGGCCCCTGGT	0.003302	3.2686
CSA033069.1	GGGCAACCCCTGCATTATCT	TGGGTCAGAGAGCCAGAGTT	0.025595	5.54529
CSA020735.1	ACTGAGTCTGATGCCCTTGC	CAGCTTGGAACCACTTTGCC	0.01306	8.02238
CSA021730.1	GTAGGTGGGGACAACGAAGG	ATGAACCATCCGGGAACACC	0.131406	11.9791
CSA016856.1	CGGGGAAAAGGAAGCCTGAT	CTTGGGCGACCACTTCTCAT	0.082188	57.3644
CSA016903.1	GAACTTGGCCTGGGCATACT	CCAGGTTGCACTGCTTGTTC	0.000668	1.51594
CSA030036.1	GCGCCAAAAGTGCAGAAAGA	GAACTGTCTTCGGCAGGTCA	0.002423	10.0673
CSA001781.1	CCCTGGAGGCATCAACCTTT	CTTGCTGGTTGGTTTGCTCC	0.000847	3.48661
CSA030020.1	GGGCAAGTGCACCAATGAAG	GGAAGGGACGCAAGATGGAA	0.001048	2.27703
CSA024443.1	GGTTTCAGCACTGGGCAAAG	CGGTGCAGAACAAATAGGCG	0.006628	4.76682
CSA007759.1	AATGAATGCTCTGGCGGTGA	TCCATCGGGGTCACAGTTTG	0.002128	12.977
CSA018589.1	TTCCTGGATTCCACCTTGCC	CCCATCTTTGAATGCCCCCT	0.000337	3.90207
CSA000647.1	GGGTGGCGATCATTGCTTTC	CTTGGTCCCCAGAGACAACC	0.000603	1.91191
CSA034653.1	CCGGAGGGTTTCACGAAGAA	AGCCACTAGTCCTTTTCCGC	0.000334	4.18459
CSA001531.1	TGGGCAGAGTGGTCGAAATC	GTGCTTTTTCAATGGCCGGT	0.014916	13.0907
CSA033342.1	CAACACCAGTTCAATGCCCG	TCAACCACCCTACGAACAGC	0.004173	20.4559
CSA021101.1	TGCTTTGTGTGCTAGAGGCA	TGTGCAGTCATCCATCCCAC	0.000947	2.91624
CSA003297.1	GTTTGTAGCTGAGGACCCCC	GAGTCTCCCTAAGGCCATGC	0.004018	5.3044
CSA002777.1	CAAGTCTACGTCCACGGCTT	CATTGCAGCGATCCTTGAGC	0.000183	2.55959
CSA018787.1	AGGAGTGAAAGCTCGGAAGC	CGGACCTCACACTTCCCTTC	0.000105	0.987532
CSA002812.1	CGGTTGTTGAGCCTGGTTTG	GAGCAGCCTCCTCACAACAT	0.006911	11.2352
CSA032127.1	ATATGGGGCTCCAAGGGAGT	GGAGGAAGGTCACAGATGGC	0.000992	2.48205
CSA030966.1	ATCACTGCGACTCCTTTGGG	GCACCTAGGCACAATCCAGT	0.000189	3.81276
CSA023919.1	TGGCTACTTGTTGCATCCGT	GGCACAAGAATGGGGACTGA	0.001625	11.5655
CSA015026.1	CGGTGTATCATCCGACAGGG	CGAAAGCCAGCACACTTGTC	0.007525	2.85557
CSA008802.1	AGGTTCACAGGCGTGTACTG	TATGCCCAGTGCCAGGAAAG	0.119118	23.2597
CSA013061.1	TCTGTGAACCAACTGCGGTA	TCCACAGCTTTCTGAGCTTG	0.000108	3.41522
CSA002792.1	TGGCACTCTCTGGAGGCTAT	TGACTGTGTCTTGCTCGTCC	0.002105	8.60674
